# PAHs from the Inside Out: Birth Outcomes Vary among Ethnic Groups in NYC

**Published:** 2008-05

**Authors:** David A. Taylor

In humans, the developmental hazards associated with exposure to urban ambient air pollution, particularly polycyclic aromatic hydrocarbons (PAHs), continue to be debated. PAHs are formed during the incomplete burning of fossil fuels, wood, and tobacco products, with high levels found in automobile exhaust and industrial emissions. This month, researchers at Columbia University report that prenatal exposure to PAHs affects birth outcomes differently in pregnant African-American and Dominican women **[*EHP* 116:658–665; Choi et al.]**.

Previously the same group showed that approximately 40% of the children studied had *in utero* DNA damage as a result of exposure to air pollution from fossil fuel combustion. The same group has also examined the link between prenatal exposure to airborne PAHs and intrauterine growth restriction, a condition in which the fetus is smaller than expected for the length of pregnancy, and has shown a relationship between prenatal exposure to PAHs and reduced birth weight in African Americans in New York City and whites in Krakow, Poland.

In the current prospective study, the team recruited women from local prenatal care clinics in New York City. The women lived in three neighborhoods served by Columbia’s Mailman School of Public Health. The target population was limited to women aged 18–35 who did not use tobacco products or illicit drugs; did not have diabetes, hypertension, or known HIV; and had started prenatal care by the twentieth week of pregnancy.

The researchers interviewed each woman in the last trimester of pregnancy, in her home, to obtain information on health, lifestyle, and exposure history. They also gave each woman a small backpack containing an air monitor for measuring PAH exposure. The personal air monitor operated continuously over a 48-hour period at 4 L per minute to simulate normal lung capacity, collecting particulate and semivolatile vapor and aerosol PAHs. Filters were replaced every 48 hours and analyzed for pyrene and carcinogenic PAHs, including benzo[*a*]pyrene (BaP), which has been associated with adverse reproductive and developmental effects in laboratory animals and humans.

The investigators collected birth outcome information from medical records, including gestational age, which was estimated by attending clinicians and validated by sonogram. The analysis included 616 mother–child pairs. The results showed universal and variable personal exposure to PAHs, with a mean exposure to BaP (0.368 ng/m^3^) consistent with the ambient BaP level in New York City.

Among African Americans, prenatal PAH exposure increased the risk of preterm delivery and the likelihood of infants being born small for gestational age (SGA; meaning birth weight is below the tenth percentile of babies of the same gestational age). As prenatal PAH exposure increased, so did cephalization index, a ratio of head circumference to birth weight that can presage developmental problems later in childhood.

Among Dominicans, there was no association between airborne PAH exposure and significant increase in risk of SGA, reduced birth weight ratio, increased cephalization index, or preterm delivery. This could reflect healthful cultural practices among recent Dominican immigrants such as more nutritious diets and more supportive social networks. The possible health-protective effects of the Dominicans’ cultural habits and the specific factors underlying the greater vulnerability of the African-American subjects warrant further study.

